# Plasma-Activated CO_2_ Dissociation to CO in Presence of CeO_2_ Mesoporous Catalysts

**DOI:** 10.3390/molecules30214312

**Published:** 2025-11-06

**Authors:** Oleg V. Golubev, Alexey A. Sadovnikov, Anton L. Maximov

**Affiliations:** 1 A.V. Topchiev Institute of Petrochemical Synthesis, Russian Academy of Sciences (TIPS RAS), 119991 Moscow, Russia; 2 Department of Chemistry, Lomonosov Moscow State University, 119991 Moscow, Russia

**Keywords:** CO_2_ utilization, plasma catalysis, ceria catalysts, dielectric barrier discharge

## Abstract

The increasing atmospheric CO_2_ concentration is one of the major environmental challenges, necessitating not only emission reduction but also effective carbon utilization. Non-thermal plasma-catalytic CO_2_ conversion offers an efficient pathway under mild conditions by synergistically combining plasma activation with catalytic surface reactions. In this study, mesoporous ceria catalysts were synthesized by different methods and characterized using N_2_ adsorption–desorption, SEM, XRD, XPS, CO_2_-TPD, and XRF techniques. The materials exhibited distinct textural and electronic properties, including variations in surface area, pore structure, and basicity. Plasma-catalytic CO_2_ dissociation experiments were conducted in a dielectric barrier discharge reactor at near-room temperature. Among the synthesized catalysts, Ce(mp)-4 demonstrated the highest CO_2_ conversion of 32.3% at a 5 kV input voltage and superior energy efficiency, which can be attributed to its meso-macroporous structure that promotes microdischarge formation and enhances CO_2_ adsorption–desorption dynamics. CO was the only product obtained, with near-100% selectivity. Catalyst stability testing showed no deactivation while spent catalyst characterization indicated carbon-containing species. The findings in this study highlight the critical role of tailored pore structure and basic-site distribution in optimizing plasma-catalytic CO_2_ dissociation performance, offering a promising strategy for energy-efficient CO_2_ utilization.

## 1. Introduction

The continuous rise in atmospheric CO_2_ concentrations, primarily driven by fossil fuel combustion and industrial activities, has become one of the defining challenges of the 21st century. Elevated CO_2_ levels intensify global warming, alter weather patterns, and threaten ecosystems and human well-being. Addressing this issue requires not only strategies to reduce emissions but also technologies capable of converting CO_2_ into valuable products, thereby closing the carbon cycle and contributing to a sustainable energy future.

Carbon capture, utilization, and storage has emerged as a promising framework to mitigate CO_2_ emissions [1]. Within this approach, CO_2_ utilization is particularly attractive, as it transforms waste carbon into fuels, chemicals, and materials, simultaneously reducing environmental burden and generating economic value [2]. However, due to the high thermodynamic stability of the CO_2_ molecule, conventional thermal catalytic processes demand high energy input and harsh conditions, limiting their efficiency and scalability [3].

Non-thermal plasma technology offers an alternative pathway of CO_2_ conversion by providing highly energetic electrons and reactive species under near-ambient conditions. These species can effectively activate the CO_2_ molecule, overcoming the intrinsic energy barrier for dissociation [4]. When combined with suitable catalysts, plasma-based systems benefit from a synergistic effect: plasma facilitates molecular activation, while catalysts enhance adsorption, lower reaction barriers, and steer selectivity towards desired products [5]. This plasma–catalyst interaction enables CO_2_ conversion at milder operating conditions compared to traditional catalytic processes, offering both energetic and environmental advantages [6,7]. It should be noted that at the current stage of plasma-catalytic CO_2_ utilization technologies, the cost of the output product and the cost of the overall process are high. For example, the cost of CH_3_OH produced by the CO_2_ hydrogenation method in plasma is many times higher than that of CH_3_OH obtained through conventional processes. The economic feasibility of CO_2_ utilization using plasma is highly sensitive to the cost of electricity, thus ensuring affordable and reliable energy sources is essential for the economic viability of this method [8]. On the other hand, the impact of plasma enables reaction pathways, which are thermodynamically unfavorable, and the plasma-driven process may be the only way to conduct such processes with a satisfactory conversion of CO_2_. Thus, plasma-enabled CO_2_ conversion cannot compete with traditional processes at this moment but has potential to be implemented in the future.

Among the different plasma sources investigated, such as microwave [9], gliding arc [10,11], and dielectric barrier discharge (DBD), DBD reactors are the most widely studied owing to their operational simplicity, low operating temperature, and adaptability for catalyst integration [12]. The choice of catalyst remains a critical factor for efficient plasma-catalytic CO_2_ dissociation and the development of a suitable catalyst is a task of great importance. For example, transition metal compounds [13,14,15], metal oxides with oxygen vacancies (e.g., CeO_2_ [16,17,18,19] and In_2_O_3_ [20,21]), and basic oxides (MgO and CaO [22,23]) have shown particular promise, as they promote CO_2_ adsorption and facilitate bond cleavage [24]. Furthermore, combining these oxides in tailored catalytic formulations may enhance performance through the coexistence of basic sites and oxygen vacancy-driven redox activity [25]. Another catalyst property, which is essential in CO_2_ dissociation, is its porosity. In the recent study, we showed that supporting CeO_2_ on mesoporous materials such as MCM-41, SBA-15, and MCF affected the performance of CO_2_ decomposition. When using wide-porous MCF-type material as a support, it was possible to achieve the highest conversion due to enhanced CO_2_ adsorption in pores and subsequent plasma-catalytic decomposition [26]. It has been shown that CeO_2_-based porous systems are promising for CO_2_ dissociation in plasma, but studies on supportless mesoporous CeO_2_ have not yet been conducted. Mesoporous CeO_2_ is a particularly promising catalyst for plasma-assisted CO_2_ conversion due to its combination of high oxygen storage capacity, redox flexibility associated with the Ce^4+^/Ce^3+^ couple, and abundant surface basic sites that promote CO_2_ adsorption and activation and other catalytic applications [27,28]. Unlike supported CeO_2_ systems, which may suffer from limited active surface exposure or support interference, self-standing mesoporous CeO_2_ offers direct access to active sites and tunable pore structure. Thus, in the current work, mesoporous CeO_2_ catalysts without SiO_2_ or Al_2_O_3_ support were synthesized using various techniques and were characterized by physico-chemical methods (N_2_ adsorption–desorption, SEM, XRD, XPS, CO_2_-TPD, and XRF).

The aim of the present study was to investigate the influence of mesoporous ceria properties (i.e., porous characteristics, basicity, and surface electron state) on CO_2_ dissociation efficiency (such as conversion, CO selectivity, and energy efficiency) in non-thermal DBD plasma. Reactions were carried out at near-room temperature in a continuous flow plasma reactor, which has the potential for future scalability.

## 2. Results and Discussion

### 2.1. Catalyst Characterization

**Figure 1:**
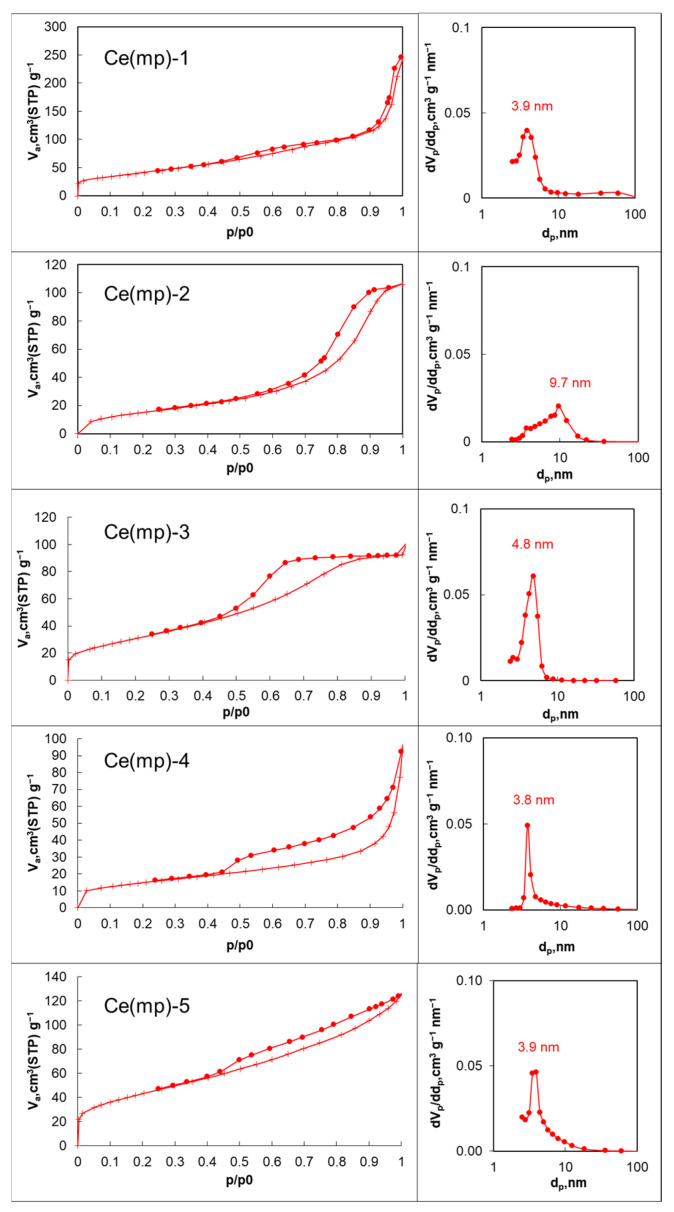
Adsorption isotherms (**left plots**) and pore size distributions (**right plots**) of the Ce(mp)-1-Ce(mp)-5 samples. Dotted line on the left plots represents desorption curve.

The synthesized catalysts were characterized by a complex of physico-chemical methods. From the low-temperature N_2_ adsorption–desorption analysis, it was revealed that the synthesized ceria samples exhibited adsorption isotherms of type IV (IUPAC) [29] with hysteresis loops of H3/H4 type [30], confirming the predominance of mesoporosity (Figure 1). However, the total adsorbed volumes and surface area values varied significantly (Table 1). The pore size distributions further indicated that the materials contained mesopores in the range of 4–11 nm, with variations in pore uniformity and total porosity depending on the synthesis conditions. It should be noted that the pore distribution peak at 3.8–3.9 nm (Ce(mp)-1, Ce(mp)-4, and Ce(mp)-5 samples) could not be taken into consideration since it was related to the adsorptive properties and not the actual porous properties of the samples [31]. Thus, it can be concluded that the named samples had rather wide pore size distributions without any actual peaks.

The combination of high surface area and mesoporosity in Ce(mp)-1 and Ce(mp)-5 suggests extensive nanoscale dispersion and interparticle void networks. Lower BET and larger pores in Ce(mp)-2 and Ce(mp)-4 indicate stronger aggregation and a coarser pore structure.

**Figure 2:**
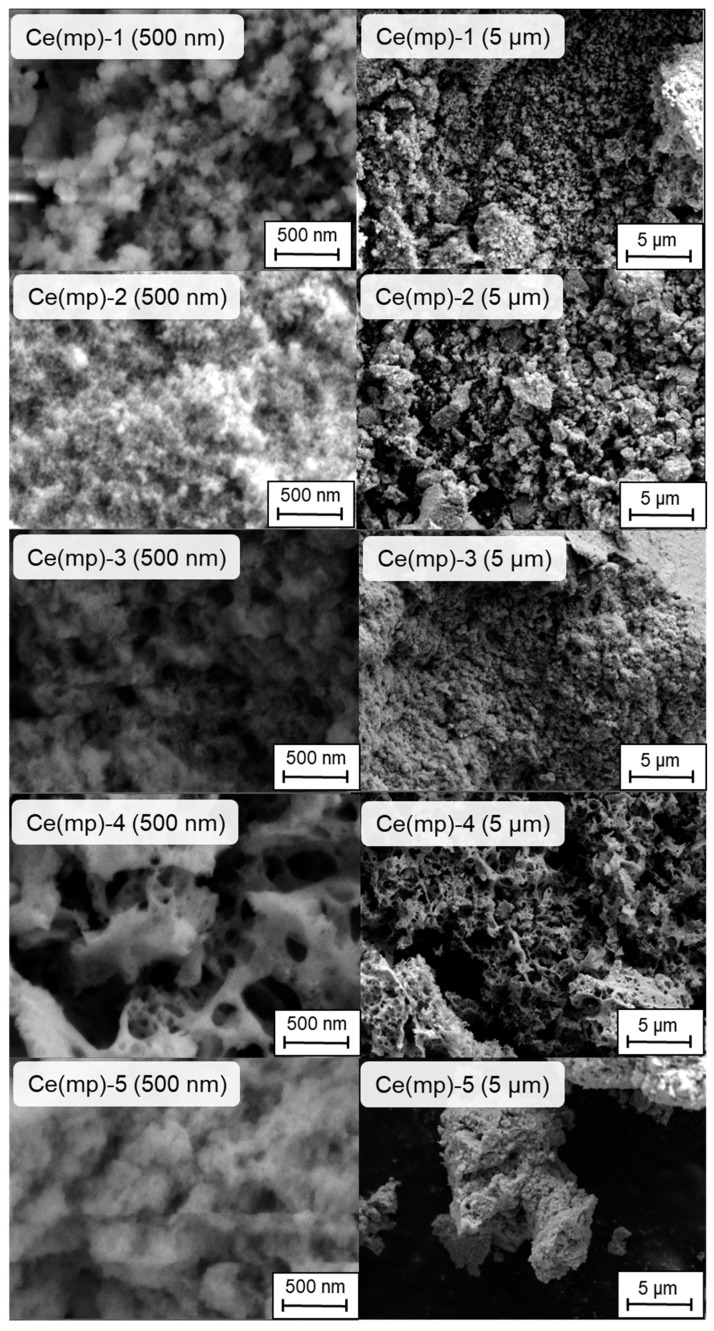
Scanning electron microscopy images of the synthesized mesoporous ceria.

To reveal the morphology of the samples, scanning electron microscopy (SEM) analysis was conducted. The microphotographs in two scales (500 nm and 5 μm) are presented in Figure 2. Ce(mp)-1 sample exhibited fine-grained, highly porous agglomerates and a dense network of connected nano-sized particles. This explained the high surface area of the sample. Ce(mp)-2 appeared as more compact and coarse aggregates, which is consistent with its large modal pore diameter and low surface area. In contrast, the Ce(mp)-3 sample had more fine particles with intermediate inter-particle pores. Ce(mp)-4 shows a notably open, sponge-like morphology with non-uniform macroporous cavities, consistent with low BET but high accessibility to the external environment. The last structure can be considered meso-macroporous, as it contained both mesopores and macropores, which can facilitate CO_2_ adsorption and its subsequent dissociation in plasma. The morphology of the Ce(mp)-5 sample was “cauliflower-like” but with a large aggregate size (which was seen at a 5 μm scale). Interestingly, the Ce(mp)-5 sample retained a relatively high adsorption capacity despite its dense morphology in SEM, suggesting the presence of internal mesopores which were not resolved at the microscale.

**Figure 3:**
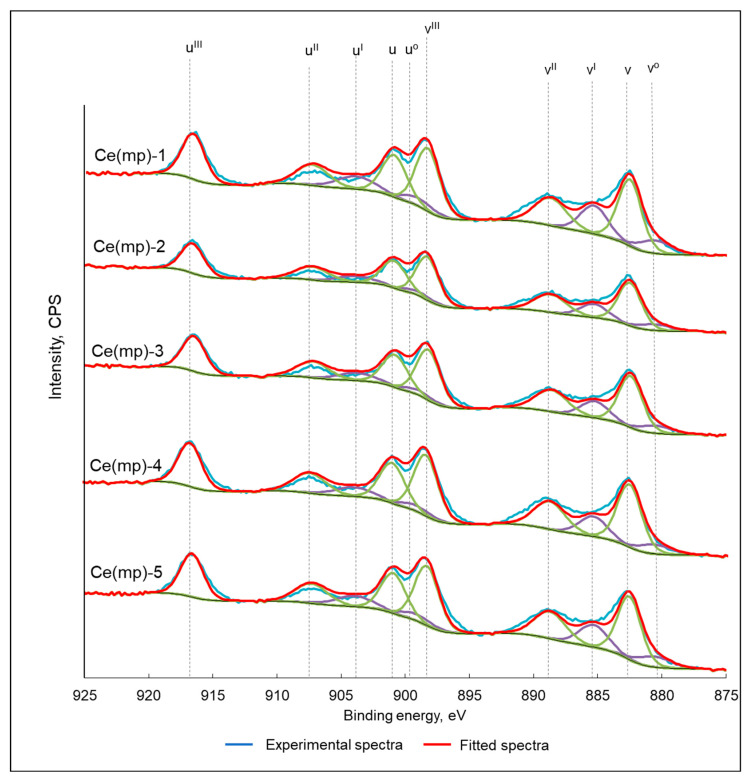
X-ray photoelectron spectra of the synthesized catalysts. Green peaks represent Ce^4+^ phase, purple peaks represent Ce^3+^ phase.

The electron state of the surface was analyzed using X-ray photoelectron spectroscopy (Figure 3). Each experimental spectrum (blue line) was deconvoluted using a series of Gaussian-Lorentzian functions to account for the multiple final states arising from Ce^3+^ and Ce^4+^ species. The resulting fitted spectra are shown as red lines, with individual components plotted in various colors (green for Ce^4+^ and purple for Ce^3+^). The Ce 3d spectrum was characteristically complex due to the mixed valence nature of cerium oxides, which resulted in the presence of multiple peaks. Ce^4+^ features are typically assigned six peaks: u’’’ (~917 eV), u’’ (~907 eV), u (~901 eV), v’’’ (~898 eV), v’’(~889 eV), and v (~882 eV) (where “u” and “v” correspond to 3d_3/2_ and 3d_5/2_, respectively). Ce^3+^ features are associated with four peaks: u’ (~904 eV), u^0^ (~899 eV), v’ (~885 eV), and v^0^ (~880 eV). The presence of Ce^3+^ was attributed to oxygen vacancies in the structure of the ceria. From the corresponding peak area, the amount of Ce^3+^ was calculated for each sample (Table 2). It can be seen that the oxygen-deficient Ce^3+^ phase was present in all the samples and its content was comparable for most Ce(mp) catalysts. The highest content of Ce^3+^ was characterized in the Ce(mp)-1 sample (23%), while Ce(mp)-4 had the lowest concentration of the partially reduced phase (17%).

**Figure 4:**
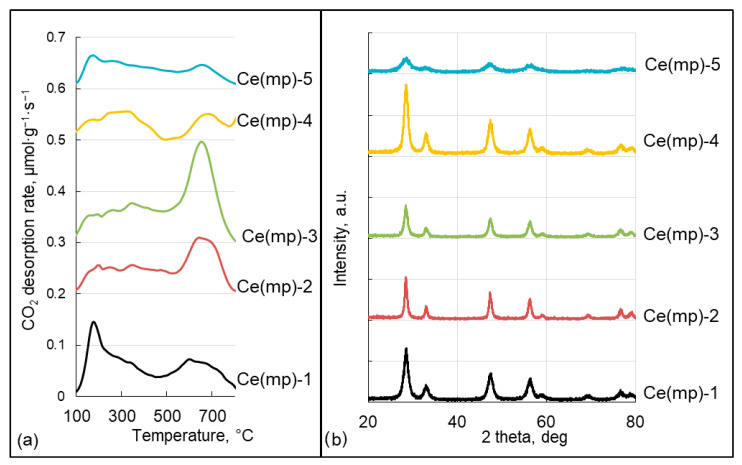
Temperature desorption profiles (**a**) and X-ray diffraction patterns (**b**) of the synthesized samples.

CO_2_-TPD profiles (Figure 4a) revealed distributions of weak (<250 °C), medium (250–400 °C), and strong (>400 °C) basic sites across the series (Table 2). Ce(mp)-3 possessed the highest total basicity (427 μmol/g) and the largest concentration of strong basic sites (282 μmol/g). Ce(mp)-1 and Ce(mp)-2 also showed substantial high-temperature desorption features (strong sites 145 and 185 μmol/g, respectively), whereas Ce(mp)-4 displayed a markedly lower total basicity (113 μmol/g) and almost no strong basic-site contribution (6 μmol/g).

X-ray diffraction patterns confirmed the presence of the cerianite CeO_2_ phase (PDF# 43-1002; 2θ = 28.5°, 33.0°, 47.5°, 56.3°, 59.1°, 69.4°, 76.7°, 79.0°) in all materials, but peak width and intensity varied between the samples (Figure 4b). Ce(mp)-1 and Ce(mp)-4 showed relatively sharp reflections indicative of larger coherent domains, while Ce(mp)-2 and Ce(mp)-3 displayed intermediate broadening. Ce(mp)-5 exhibited the most broadened and weak reflections, which indicated either small crystallite size or high dispersion of ceria particles. Crystallite-size analysis with XRD yielded average domain sizes of 3–14 nm (Table 2): Ce(mp)-5 was the least crystalline with the smallest average crystallite size (3 nm), whereas Ce(mp)-2 exhibited the largest coherent domain size (14 nm).

### 2.2. CO_2_ Dissociation Experiments

**Figure 5:**
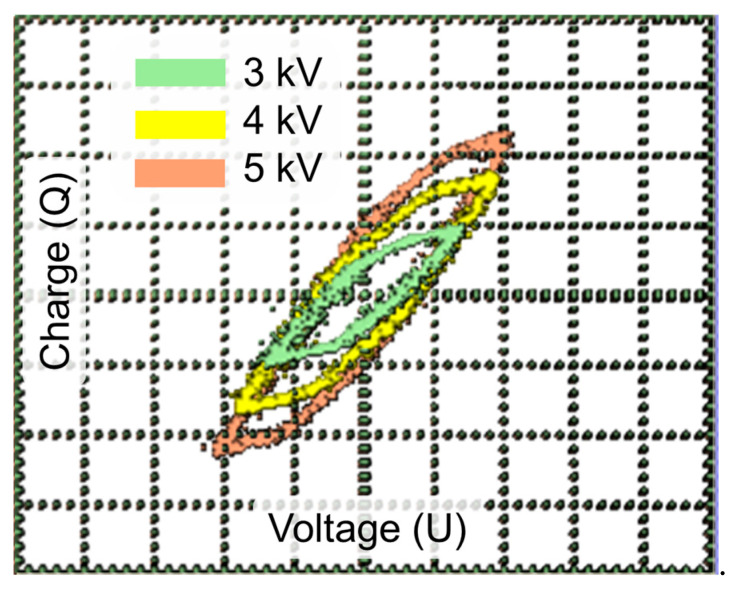
Lissajous figures captured in plasma-only mode.

Lissajous figures, which were captured in plasma-only mode, are presented in Figure 5. The shape of the figure is a parallelogram, which is typical for DBD plasma [32,33]. It can be seen that, with increasing input voltage, the shape of the figure is extended along the “Charge” axis, which can be explained by the increase in the current (from ~15 mA to ~34 mA) and thus the increase in absorbed plasma power.

**Figure 6:**
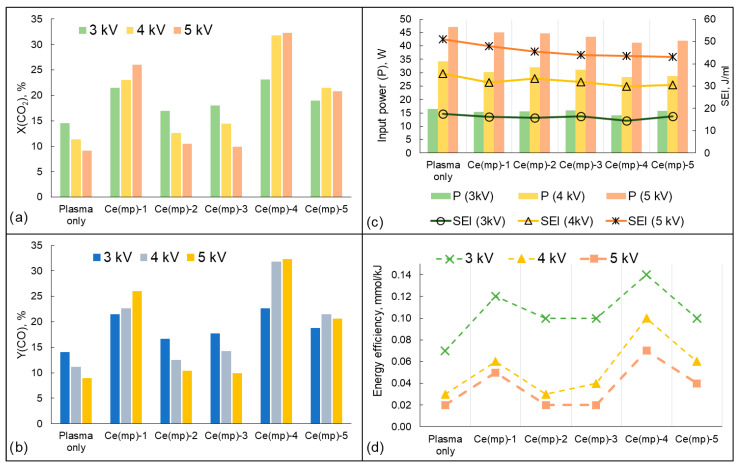
The results of plasma-only and plasma-catalytic CO_2_ dissociation: (**a**) CO_2_ conversion; (**b**) CO yield; (**c**) input power and specific energy input; (**d**) energy efficiency.

The experiments on plasma-catalytic CO_2_ dissociation were conducted using obtained CeO_2_ catalysts (Ce(mp)-1—Ce(mp)-5) and with variations in the input voltage. The results of the experiments are presented in Figure 6.

When conducting plasma-only experiments, it was observed that, with increasing input power, CO_2_ conversion was lowered. The maximum achieved conversion in the absence of the catalyst was 14.5% at an input power of 15 W (3 kV of the input voltage). When the power was increased up to 34 W (4 kV input voltage), the conversion tended to decrease to 11.5%, with a further decrease to 9.1% at 45 W (5 kV input voltage). The observed trend could be related to the existence of the back reaction (CO + 1/2O_2_ → CO_2_), which restricts further conversion of CO_2_ in the absence of the catalysts. It was noteworthy that a similar CO_2_ conversion trend was observed in the presence of the Ce(mp)-2 and Ce(mp)-3 catalysts. When these catalysts were loaded into the DBD reactor, the conversion of CO_2_ was slightly higher than that in the plasma-only reactor at an input voltage of 3 kV and 4 kV, but when a 5 kV voltage was applied, the change in conversion was nonsignificant (compared to the plasma-only mode). Another pattern was observed when the Ce(mp)-1, Ce(mp)-4, and Ce(mp)-5 samples were placed into the reactor. The conversion of CO_2_ increased with increasing input voltage up to 5 kV. However, the energy efficiency value dropped at 5 kV, as the input power value and specific energy input were enhanced, with a simultaneous decrease or slight increase in the CO_2_ conversion (Figure 6d). Thus, the 5 kV mode can be considered as the least energy-effective mode for the CO_2_ dissociation reaction in the studied conditions.

The highest CO_2_ conversion was achieved using the Ce(mp)-4 catalyst (32.3% at 5 kV), and the energy efficiency of the CO_2_ dissociation process was also the highest among the samples in the presence of the Ce(mp)-4 sample (Figure 6d). From the results of the experiments, it was observed that CO was the only product of CO_2_ dissociation and the selectivity of CO was close to 100% in all the experiments. Consequently, the yield of CO was nearly the same as the CO_2_ conversion (Figure 6b).

In the presence of the samples Ce(mp)-1 and Ce(mp)-5, a similar CO_2_ conversion was observed at a 4 kV input voltage, which was attributed to their comparable properties. For instance, the specific surface area of the Ce(mp)-1 sample was 150 m^2^/g, while that of the Ce(mp)-5 sample was 155 m^2^/g (Table 1). However, due to the higher number of electronic defects on the surface and the greater concentration of weak basic sites (Table 2), a higher degree of CO_2_ conversion was achieved in the presence of the Ce(mp)-1 sample.

**Figure 7:**
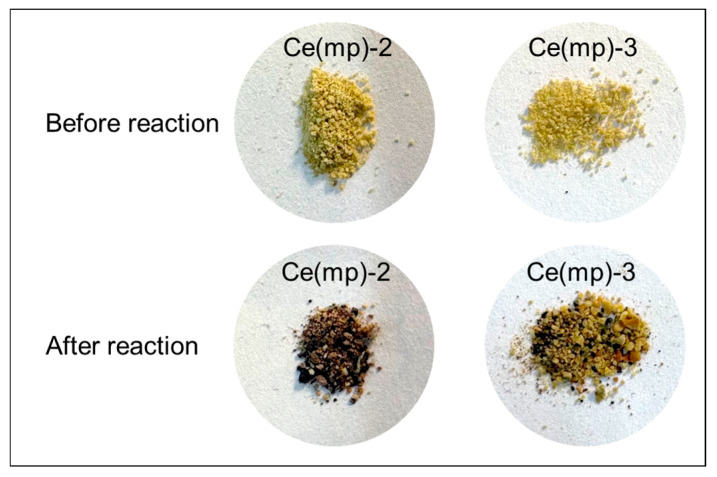
Photographs of the Ce(mp)-2 and Ce(mp)-3 catalysts before and after the plasma-catalytic reaction.

From the results of the plasma-catalytic experiments, it was mentioned that the Ce(mp)-2 and Ce(mp)-3 samples had minor CO_2_ dissociations, in comparison with other obtained samples. The samples after the experiment changed their color (Figure 7) to a mixture of the initial yellow with dark brown and black particles. Apparently, due to the synthesis procedure (without calcination stage), cetyltrimethyl ammonium bromide was not fully removed from the initial mixture and, in the DBD medium, was partially decomposed, as was shown in [34]. A detailed characterization and discussion of catalysts after the reactions are provided in Section 2.3.

Furthermore, samples with a high concentration of strong basic sites, such as Ce(mp)-2 and Ce(mp)-3, exhibited limited CO_2_ conversion despite their overall high basicity. This suggests that excessively strong adsorption can hinder CO_2_ desorption under plasma conditions, limiting the catalytic turnover. In contrast, Ce(mp)-4, which possesses predominantly medium-strength basic sites and low total basicity, showed superior conversion and energy efficiency. The moderate basicity facilitates sufficient CO_2_ adsorption while allowing for effective desorption of the reaction products, enabling continuous catalytic activity. Ce(mp)-1 and Ce(mp)-5, with intermediate distributions of weak and medium sites, demonstrated performance between these extremes. Overall, these results highlight that not just the total number of basic sites but also their strength distribution critically determine the efficiency of plasma-catalytic CO_2_ dissociation. As it was revealed in our previous work [25], the presence of strong basic sites in CO_2_ dissociation catalysts may play a negative role, as CO_2_ cannot be desorbed under the reaction conditions. Similar observations were obtained when comparing MgO and CaO samples for CO_2_ sorption in [35].

To provide a quantitative comparison of the activity of the CeO_2_ catalysts and clarify the role of surface basicity, the apparent turnover frequencies (TOF) for CO formation were calculated for each sample. The TOF values were calculated from the CO formation rates and the number of surface Ce atoms estimated from the CeO_2_ (111) surface density (7.9 Ce atoms per nm^2^ [36,37]), BET surface areas, and Ce^3+^ concentration obtained from XPS, representing the assumed number of catalytically active sites. The detailed calculation procedure is given in the Supplementary Materials. Despite having the lowest surface area (52 m^2^/g), the Ce(mp)-4 catalyst exhibited the highest calculated TOF (0.0225 s^−1^), indicating that its active Ce sites were intrinsically the most efficient (Table 3). CO_2_-TPD analysis showed that Ce(mp)-4 possessed predominantly weak and medium basic sites with almost no strong sites, whereas samples with higher total basicity or stronger sites (Ce(mp)-1, Ce(mp)-3, Ce(mp)-5) exhibited lower TOFs. This inverse correlation between strong basicity and TOF confirms that moderate basicity yields optimal adsorption–desorption kinetics, providing sufficient CO_2_ activation without inhibiting CO desorption.

**Figure 8:**
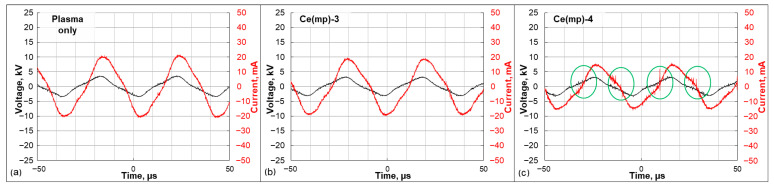
Voltage and current oscillograms captured during the CO_2_ plasma-catalytic dissociation at a 4 kV input voltage: (**a**) plasma-only reactor; (**b**) reactor packed with the Ce(mp)-3 sample; (**c**) reactor packed with the Ce(mp)-4 sample.

It can be observed that the developed meso-macroporous structure of Ce(mp)-4 was beneficial for the CO_2_ dissociation reaction in plasma. The alteration of DBD physical characteristics was observed in the presence of the Ce(mp)-4 catalyst. In Figure 8 voltage–current oscillograms are presented for the plasma-only and Ce(mp)-3-and Ce(mp)-4-loaded experiments. It can be seen that the current oscillogram in the plasma-only experiment is relatively smooth without any sharp peaks. When the catalyst was present (Figure 8b), the amplitude of the current oscillogram decreased due to catalyst packing, which reduced the free volume of the reactor. When the Ce(mp)-4 sample was packed into the reactor, the amplitude of the oscillogram also decreased due to the high volumetric density of the sample. Distinguishable peaks are clearly seen on the current oscillogram (marked in green circles, Figure 8c), corresponding to microdischarges, which were likely to appear in the meso-macroporous structure of the Ce(mp)-4 catalyst. It is reported in the literature that the microdischarges that were formed inside of the catalyst pores had a size of 4–100 nm and wider pores with sizes at the μm level [38]. Such microdischarges enhance the local electric field and thus increase the CO_2_ conversion.

**Figure 9:**
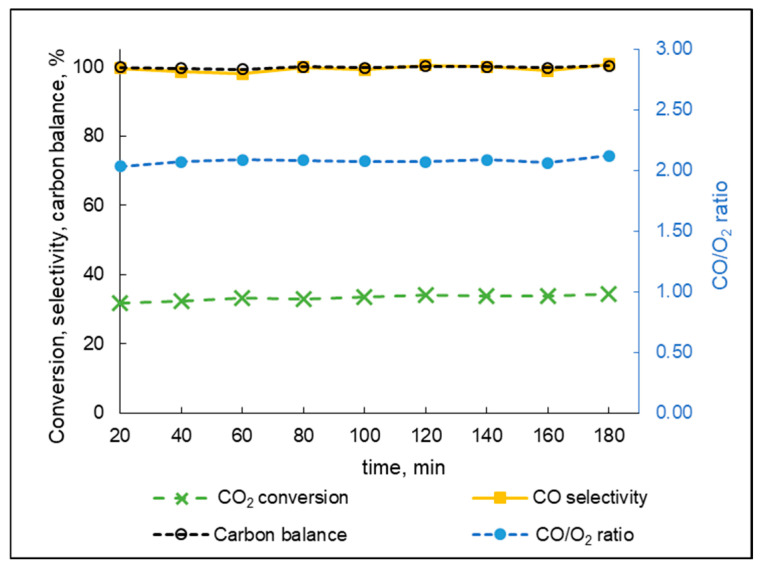
Catalyst stability test during 180 min of constant CO_2_/Ar flow at 4 kV input voltage.

The test on catalyst stability was conducted using the Ce(mp)-4 sample as the most active catalyst within the series. The results of the experiment are presented in Figure 9. Within the measured time, the catalyst did not tend to deactivate, as no drop in conversion was observed. In contrast, an increase in CO_2_ conversion was recorded from 31.7% (20 min of runtime) to 34.3% (180 min of runtime). Furthermore, CO selectivity and carbon balance values were close to 100% and the CO/O_2_ ratio was close to 2.0, which suggested that no undesirable products like solid carbon were produced.

The obtained results were compared with the recently published ones on plasma-catalytic CO_2_ dissociation and were summarized in Table 4. Reported CO_2_ conversions ranged from about 9 to 52%, with energy efficiencies between 0.1 and 1.5 mmol CO per kJ, depending on reactor design and catalyst type. Low-power DBD systems generally achieved higher energy efficiency but lower CO_2_ conversion, while higher-power configurations led to higher conversion. Cerium-based catalysts consistently showed enhanced performance, likely due to the redox activity of Ce species and the presence of oxygen vacancies, which facilitate CO_2_ dissociation. However, the operating conditions in the reviewed studies varied significantly in terms of reactor geometry, discharge power, gas composition, and flow rate, which complicates a direct quantitative comparison of the results. It can also be seen that combining the right catalyst choice and special techniques (e.g., electrode cooling) helps increase conversion drastically. Nevertheless, the CeO_2_ mesoporous catalyst developed in this work shows one of the highest conversions under comparable discharge powers, highlighting the beneficial role of the CeO_2_ surface and meso-macroporous structure in promoting CO_2_ splitting efficiency.

### 2.3. Catalyst Characterization After the Reaction

**Figure 10:**
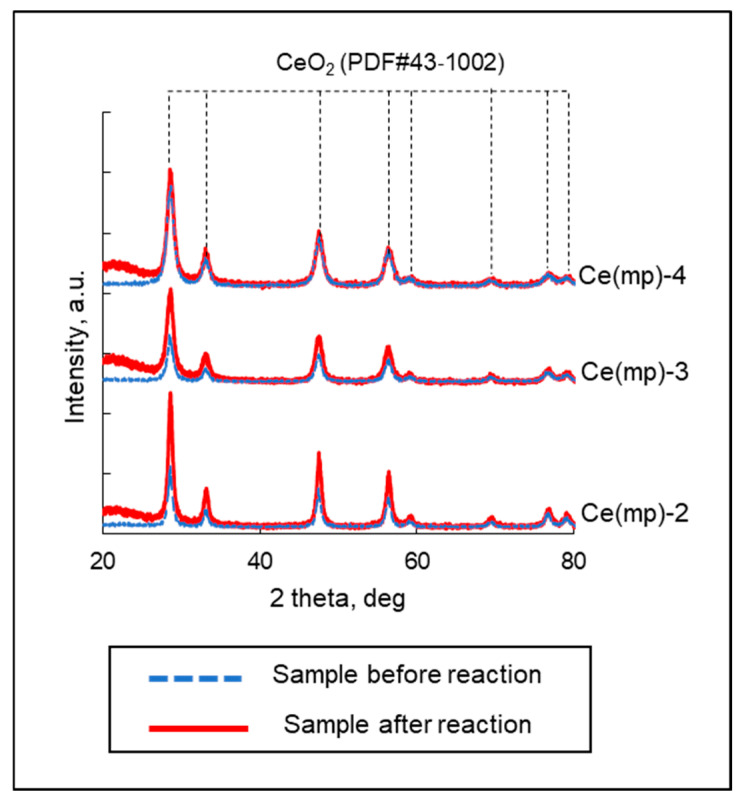
XRD analysis of the samples after reaction.

The catalysts (Ce(mp)-2–Ce(mp)-4) were characterized after the reactions to estimate changes in their physico-chemical characteristics. From XRD analysis, it was observed that no additional phases were present on the diffractograms after the reactions (Figure 10). The calculated average size of the crystallites (Table 5) was close to the crystallite size before the reactions for all samples (Table 2). This indicated that the nanostructure of the catalysts remained stable and agglomeration of the crystallites did not occur. From the low-temperature adsorption–desorption analysis, it was seen that the textural properties of the samples remained practically unchanged, and preservation of the mesoporous structure was evident from the adsorption isotherms (Figure S1). Additionally, XPS analysis did not reveal any drastic changes in the surfaces of the spent samples (Figure S2). It can be noted that the Ce^3+^ content increased (from 18 to 22%) only in the Ce(mp)-2 and Ce(mp)-3 samples, suggesting the redox properties of the ceria slightly changed under plasma conditions.

**Figure 11:**
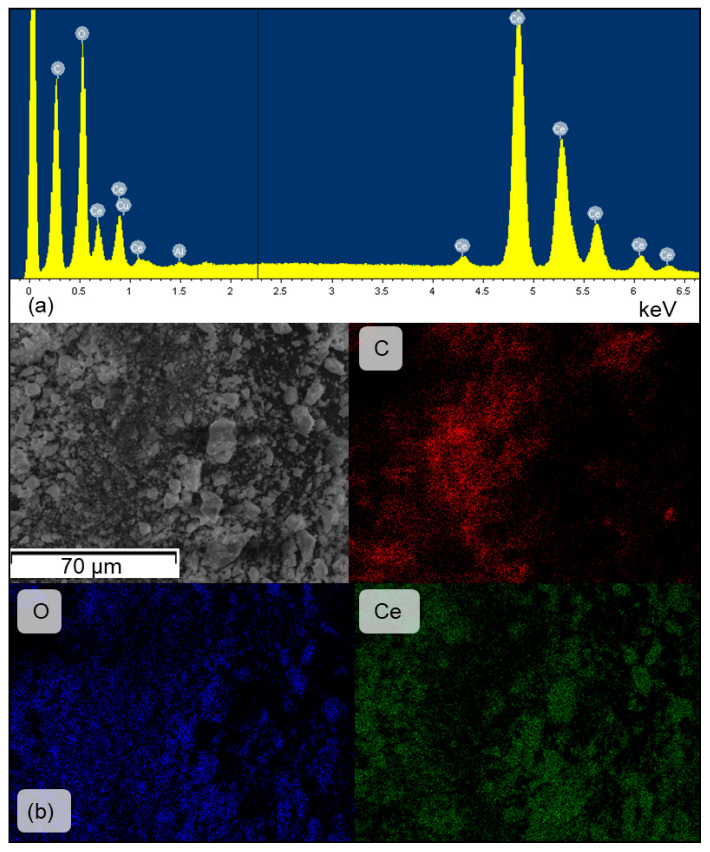
SEM-EDX analysis of the Ce(mp)-2 spent sample: (**a**) EDX spectrum; (**b**) elemental mapping.

The morphology and composition of the catalyst surface after reaction were additionally analyzed by SEM–EDX and compared with TGA results. As shown in Figure 11, the SEM micrograph reveals aggregates of irregularly shaped CeO_2_ particles with dimensions in the submicron-to-several-micron range. The morphology remains largely preserved, indicating that plasma exposure did not cause noticeable sintering or fragmentation. The corresponding EDX spectrum shows intense signals from Ce and O, consistent with the presence of CeO_2_ as the main phase (Figure 11a) with an additional carbon peak. Elemental mapping (Figure 11b) shows a homogeneous distribution of Ce and O and a clear presence of C on the surface of the sample. The localized character of the carbon signal suggests surface deposition rather than bulk incorporation, and its intensity exceeds the level typically attributed to carbon coating or atmospheric contamination. It is also revealed from the EDX analysis that the highest carbon concentration on the sample surface occurred with the Ce(mp)-2 and Ce(mp)-3 samples, which may support the assumption of incomplete CTAB decomposition during the synthesis. However, it can also be seen that Ce(mp)-4 contained carbon surface atoms as well, but to a lesser extent.

The pointed findings correlate with the TGA results (Figures S3–S5), which revealed a small mass loss of 2–4% accompanied by a DTG peak at 330–335 °C for all samples. This temperature range coincides with the thermal decomposition of cerium (III) oxalate, which typically decomposes to CeO_2_ with the release of CO and CO_2_ in an air atmosphere [43]. Formation of oxalate species, however, has not been reported in the literature for the related conditions but may be explained by the redox behavior of CeO_2_ under plasma conditions. The plasma environment generates a high density of reactive CO_2_ fragments (CO, CO_2_^–^, and CO_x_ radicals) that can interact with reduced Ce^3+^ surface sites. As previously stated for thermocatalytic and photocatalytic systems [44,45], these interactions promote the coupling of CO_2_-derived species to form adsorbed carbon–oxygen intermediates, including oxalates. Subsequent thermal oxidation of these intermediates regenerates CeO_2_ and releases CO and CO_2_, consistent with the TGA mass-loss observations.

Therefore, while carbon was found by means of SEM-EDX, it cannot have been related to solid carbon deposition from the decomposition of CO_2_ to C and O_2_ during the plasma-catalytic reaction. Either the carbon originated from an incomplete precursor decomposition or a newly formed species like oxalate intermediates. The CeO_2_ lattice remained structurally stable based on the XRD analysis, while surface atoms may have facilitated reversible storage and oxidation of these species during plasma exposure. The latter assumption needs to be carefully checked and confirmed by means of in situ/operando methods.

## 3. Materials and Methods

### 3.1. Catalyst Synthesis

For the synthesis of the mesoporous ceria catalysts, various techniques were used. A brief summarization of the obtained ceria samples is presented in Table 6 and a detailed description of the synthesis is given below.

The synthesis of the Ce(mp)-1 sample was carried out using the technique [46] as a reference. In a typical synthesis, 5.21 g of Ce(NO_3_)_3_·6H_2_O (99%, LLC “Tsentr Tekhnologii Lantan”, Moscow, Russia) was dissolved in 84 cm^3^ of distilled water to obtain a clear solution. Then, 3.79 g of NH_4_HCO_3_ (pure, LLC “Spektr-Him”, Moscow, Russia) was added to the solution under vigorous stirring and the white precipitate Ce_2_(CO_3_)_3_ was produced. After that, 21 cm^3^ of H_2_O_2_ (37 vol% solution, LLC “Rushim.ru”, Moscow, Russia) was added dropwise to the suspension and the color of the precipitate turned orange. The suspension was stirred for 30 min and subsequently aged for 3 h without stirring. The mixture was transferred into a 150 cm^3^ stainless steel Teflon-lined autoclave and aged at 200 °C for 24 h. The product was then filtered, washed thoroughly with distilled water, and dried in an oven to obtain the Ce(mp)-1 sample.

The synthesis of the Ce(mp)-2 and Ce(mp)-3 samples was carried out using the technique [47] as a reference. Briefly, 3.9 g of Ce(NO_3_)_3_·6H_2_O and 1.09 g (2.18 g in case of Ce(mp)-3 sample) of cetyltrimethylammonium bromide (CTAB, >99%, HiMedia, Thane, India) were dissolved in 120 cm^3^ of distilled water. After the dissolution of the reagents, 12 cm^3^ of aqueous ammonia (NH_3_·H_2_O, pure for analysis, LLC “Sigma-Tek”, Khimki, Russia) was added dropwise to the solution, after which it was transferred into a 150 cm^3^ stainless steel Teflon-lined autoclave and aged at 100 °C for 24 h. The product was then filtered, washed thoroughly with distilled water, and dried in an electric oven.

The synthesis of the Ce(mp)-4 sample was carried out in accordance with the technique [48] using citric acid (JSC “LenReactiv”, Saint-Petersburg, Russia) as a pore-developing agent.

The synthesis of the Ce(mp)-5 sample was carried out using the technique [49] as a reference. In a typical synthesis, 6 g of NaOH (pure for analysis, JSC “LenReactiv”, Saint-Petersburg, Russia) was dissolved in 160 cm^3^ of distilled water. Simultaneously, 9.76 g of Ce(NO_3_)_3_·6H_2_O was dissolved in 20 cm^3^ of distilled water and then the solution was added dropwise to the NaOH solution and a purple precipitate was produced. The mixture was stirred for 2 h and then the precipitate was washed with distilled water until it became a neutral medium. The precipitate was dried in air and then in an oven at 60 °C for 9 h.

### 3.2. Physico-Chemical Methods

X-ray diffraction (XRD). XRD patterns were recorded using the TD-3700 X-ray diffractometer (Tongda Science & Technology Co., Ltd., Dandong City, China). The device was equipped with a copper anode X-ray tube, a Mythen2R 1K linear multichannel semiconductor detector, and a Goebel mirror for parallel beam formation. Data were collected over a 2θ range of 10–90°. Average crystallite size was estimated using Williamson–Hall analysis [50].

N_2_ adsorption–desorption. The specific surface area (S_BET_), pore volume (V_pores_), and pore diameter (d_pores_) of the catalysts were measured using the BELSORP MINI X analyzer (Microtrac MRB, Osaka, Japan). Prior to analysis, samples underwent thermal degassing at 300 °C for 8 h under a pressure of 10 Pa. The Brunauer–Emmett–Teller (BET) method was employed to calculate the specific surface area within a relative pressure range (p/p_0_) of 0.05–0.2.

X-ray photoelectron spectroscopy (XPS). The properties of surface Ce atoms were determined by X-ray photoelectron spectroscopy on a PREVAC EA15 spectrometer (PREVAC, Rogów, Poland) with a high-resolution hemispherical analyzer. Characteristic non-monochromatized Al Kα X-ray emission (hν = 1486.6 eV, 150 W) was applied during the analysis. The binding energy scale was calibrated to the position of the photoelectron lines of the ground levels of gold (Au4f7/2—84.0 eV) and silver (Ag3d5/2—368.3 eV). The spectral data were analyzed using CasaXPS software (Version 2.3.24PR1.0).

Scanning electron microscopy. The surface morphology and elemental analysis of the samples were examined using scanning electron microscopy (SEM) combined with energy dispersive X-ray spectroscopy (EDX) with a NVision 40 (Carl Zeiss, Oberkochen, Germany) microscope equipped with an Oxford Instruments X-Max EDX detector (Oxford Instruments, Carl Zeiss, Oberkochen, Germany) operated at 20 kV.

X-ray fluorescence spectroscopy (XRF). The elemental composition of the catalysts was determined using the ARL PERFORM’X Sequential Spectrometer (Thermo Fisher Scientific, Ecublens, Switzerland) with an X-ray tube power of 2500 W.

CO_2_-Temperature programmed desorption (CO_2_-TPD). The basic properties of the catalysts were evaluated using the CO_2_-TPD method with USGA-101 (LLC “Unisit”, Moscow, Russia) equipment. The catalyst sample was treated at 512 °C for 40 min to remove water and oxygen, and then exposed to CO_2_ at 60 °C for 24 min. The adsorbed CO_2_ was subsequently removed at 102 °C and the CO_2_ desorption was analyzed using a thermal conductivity detector.

Thermogravimetric analysis (TGA) with differential scanning calorimetry was conducted using the TGA/DSC 3+ analyzer (Mettler Toledo, Columbus, OH, USA) at a temperature range of 30–900 °C in an air atmosphere.

### 3.3. Plasma-Catalytic Experiments

**Figure 12:**
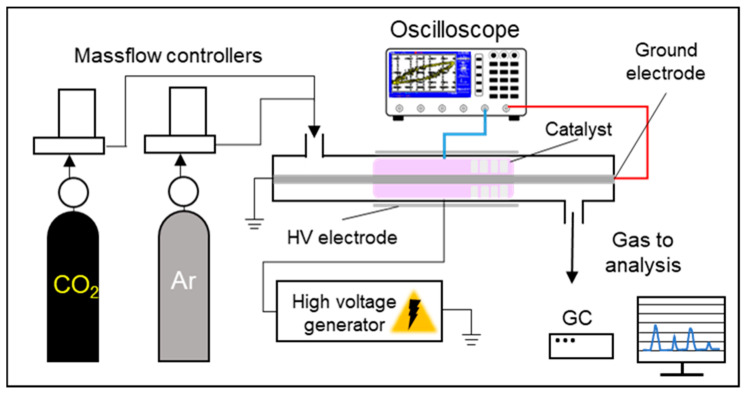
A scheme of the plasma-catalytic unit for CO_2_ dissociation.

The experiments on CO_2_ dissociation were performed using a plasma-catalytic setup equipped with a DBD reactor (Figure 12). The system consisted of a gas supply unit, the DBD reactor, a high-voltage power source, and measurement devices. The reactor body, serving also as the dielectric barrier, was a quartz tube (16 mm outer diameter, 2 mm wall thickness, and 160 mm length). A threaded steel rod (8 mm diameter) placed inside the tube acted as the grounded electrode, while a steel mesh (0.2 mm mesh size and 80 mm length) wrapped around the outside functioned as the high-voltage electrode. The discharge gap was 4 mm.

The catalyst (1 g) was loaded into the reactor and fixed between two layers of quartz wool. The particle size slightly varied depending on the catalyst, but was within the range of 0.1–0.5 mm. A CO_2_–Ar gas mixture was supplied through mass flow controllers (RRG-20, LLC “Eltochpribor”, Zvenigorod, Russia). For all experiments, the CO_2_ flow rate was set to 12 mL/min, with a CO_2_:Ar ratio of 20:80 (vol.%). The high-voltage source provided a sinusoidal signal at 25 kHz. Discharge voltage and current were monitored with a TDS 2012B oscilloscope (Tektronix, Beaverton, OR, USA) using a capacitive voltage divider circuit. Experiments were conducted within an input voltage range of 3–5 kV and a current range of 15–34 mA. Plasma-absorbed power, determined by the Lissajous figure method, varied between ~15 and ~45 W depending on the input voltage.

Reaction products were analyzed using a gas chromatograph PIA (LLC “NPF MEMS”, Samara, Russia) equipped with a thermal conductivity detector, a Hayesep N column (2 m), and a molecular sieve 13 Å column (2 m).

The performance of the process was evaluated using the following equations.

Conversion of CO_2_ was calculated as

(1) $$X\left({CO}_{2}\right)\left(\%\right)=\frac{{\nu ({CO}_{2})}_{inlet}-{\nu ({CO}_{2})}_{outlet}}{{\nu ({CO}_{2})}_{inlet}}\times 100\%$$

where *ν*(*CO*_2_)*_inlet_* is the quantity of CO_2_ which is put into the reactor (mol), and *ν*(*CO*_2_)*_outlet_*is the quantity of CO_2_ flowing out of the reactor (mol).

Yield of CO was calculated as

(2) $$Y\left(CO\right)\left(\%\right)=\frac{\nu (CO)}{{\nu ({CO}_{2})}_{inlet}}\times 100\%$$

Selectivity of CO was calculated as

(3) $$S\left(CO\right)\left(\%\right)=\frac{\nu (CO)}{{\nu ({CO}_{2})}_{conv}}\times 100\%$$

where *ν*(*CO*_2_)*_conv_* is the quantity of CO_2_ which was converted during plasma-catalytic dissociation.

Plasma-absorbed power was calculated from the Volt–Coulomb characteristic (Lissajous figure) of the discharge as follows [51]:

(4) $$P=fW=f\int_{t_{0}-\frac{T}{2}}^{t_{0}+\frac{T}{2}}u\left(t\right)i\left(t\right)dt=fC_{n}\int_{t_{0}-\frac{T}{2}}^{t_{0}+\frac{T}{2}}u\left(t\right)du_{c}\left(t\right)=fC_{n}A$$

where *u*(*t*) is the discharge voltage, *i*(*t*) is the discharge current, *C_n_* is the value of the capacitor included in series with the discharge tube, *u_c_*(*t*) is the voltage on the *C_n_*, *T* is the period of the applied voltage, *f* is the frequency of the applied voltage, and *A* is the area of the Lissajous figure.

Energy efficiency of the process was calculated as follows:

(5) $$\eta \left(\frac{mmol}{kJ}\right)=\frac{X\left({CO}_{2}\right)}{P}\times \frac{1000}{60}.$$

Specific energy input (SEI) was calculated as:

(6) $$SEI\left(\frac{J}{ml}\right)=\frac{P\times 60}{flowrate\;(\frac{ml}{min})}.$$

Carbon balance was calculated as:

(7) $$CB\left(\%\right)=\frac{{\nu ({CO}_{2})}_{outlet}+\nu (CO)}{{\nu ({CO}_{2})}_{inlet}}.$$

## 4. Conclusions

Dissociation of CO_2_ in plasma in the presence of mesoporous ceria catalysts was conducted. This study demonstrates that the physico-chemical properties of mesoporous CeO_2_ catalysts strongly influence their performance in CO_2_ dissociation under DBD plasma. Specifically, Ce(mp)-4, characterized by a meso-macroporous structure and moderate basicity, led to the highest CO_2_ conversion and energy efficiency. Its structure favored microdischarge formation inside the pores, enhancing plasma-catalyst interactions and promoting CO_2_ activation and desorption. In contrast, catalysts with excessive strong basic sites or dense morphology showed lower activity due to limited CO_2_ desorption or insufficient plasma-catalyst synergy. The Ce(mp)-4 catalyst also exhibited remarkable stability over extended operation without solid carbon deposition or deactivation. Our findings related to spent catalyst characterization suggest the preservation of the mesoporous structure and bulk phase composition; however, carbon species were found and were related either to incomplete precursor decomposition or to intermediate carbon species (e.g., oxalate) formation in a plasma environment. These results provide insight into the design principles of plasma-catalytic materials and suggest that tailored pore structures and controlled basic-site distribution are key factors for efficient CO_2_ conversion.

This study contributes to the development of scalable, low-temperature CO_2_ utilization technologies. Future studies will address the optimization of reaction parameters, including reactor design, plasma power, and CO_2_ flowrate and their influence on process efficiency. Another important goal will be to decrease the ceria loading while maintaining high CO_2_ conversion.

## Figures and Tables

**Table 1:** Textural characteristics of the synthesized catalysts.

Sample	S_BET_, m^2^/g	V_pores_, cm^3^/g	d_pores_, nm
Ce(mp)-1	150	0.34	10.7
Ce(mp)-2	58	0.16	9
Ce(mp)-3	113	0.14	4.5
Ce(mp)-4	52	0.11	9.5
Ce(mp)-5	155	0.19	5.6

**Table 2:** Physico-chemical properties of the samples: basic site concentration, revealed from CO_2_-TPD analysis; average crystallite size, calculated from XRD analysis; Ce^3+^ concentration, calculated from XPS data; Ce total content, calculated from XRF analysis.

Sample	Basic-Site Concentration, μmol/g				Average Crystallite Size, nm	Ce^3+^ Concentration, %	Ce Content, %
	Weak (<250 °C)	Medium (~250–400 °C)	Strong (>400 °C)	Total			
Ce(mp)-1	97	85	145	327	7	23	79.6
Ce(mp)-2	45	68	185	298	14	18	79.8
Ce(mp)-3	52	93	282	427	9	18	80.0
Ce(mp)-4	42	65	6	113	9	17	80.4
Ce(mp)-5	52	67	100	219	3	20	80.3

**Table 3:** Estimated TOFs for the plasma-catalytic CO_2_ dissociation at 4 kV in the presence of the mesoporous ceria catalysts.

Sample	TOF, s^−1^
Ce(mp)-1	0.0042
Ce(mp)-2	0.0075
Ce(mp)-3	0.0044
Ce(mp)-4	0.0225
Ce(mp)-5	0.0043

**Table 4:** Comparison of the plasma-catalytic CO_2_ decomposition performance with data from the literature.

Catalyst	Total Flowrate mL/min	Input Power, W	Energy Efficiency, mmol/kJ	X(CO_2_), %	Ref.
15%NiO/γ-Al_2_O_3_	30	1.8	1.13	9	[14]
CeO_2_ coated on quartz	10	27	–	26	[17]
CeO_2_—cubic	10	15–20	–	16	[18]
CeO_2_—hexagonal				20	
CeO_2_/MCF-Al_2_O_3_	60	15	0.1	19	[26]
SrO/γ-Al_2_O_3_	30	1.8	1.46	12	[39]
5 wt.% Fe_2_O_3_—5 wt.% CeO_2_/Al_2_O_3_	40	15	–	24.5	[40]
10 wt.% CeO_2_/Al_2_O_3_			–	28.5	
Ni/Al_2_O_3_	50	17	–	24	[41]
Ni/Al_2_O_3_ (with electrode cooling)		13.5	–	52	
TiO_2_ glass beads	500	1.9	–	11.5	[42]
Ce(mp)-4 (mesoporous ceria)	60	28	0.1	32	This work

**Table 5:** Physico-chemical properties of the Ce(mp)-2–Ce(mp)-4 samples after the reaction: textural characteristics, revealed from N_2_ adsorption–desorption analysis; average crystallite size, calculated from XRD analysis; Ce^3+^ concentration, calculated from XPS data; atomic distribution on the surface, calculated from SEM-EDX analysis.

Sample	Textural Characteristics			Ce^3+^ Concentration, %	Average Crystallite Size, nm	Atomic Distribution on the Surface, %					
						C		O		Ce	
	S_BET_, m^2^/g	V_pores_, cm^3^/g	d_pores_, nm			at	wt	at	wt	at	wt
Ce(mp)-2 (spent)	70	0.18	9	22	13	58	29	32	21	9	50
Ce(mp)-3 (spent)	112	0.19	6	22	9	31	9	49	20	20	71
Ce(mp)-4 (spent)	52	0.12	10	16	9	17	4	58	20	25	76

**Table 6:** Designation of the catalysts and a brief description of synthesis methods.

Sample	Preparation Method
Ce(mp)-1	Ce_2_(CO_3_)_3_ oxidation	
Ce(mp)-2	Ce(NO_3_)_3_ + CTAB + NH_4_OH	
Ce(mp)-3		
Ce(mp)-4	Ce(NO_3_)_3_ + citric acid	
Ce(mp)-5	Ce(NO_3_)_3_ + NaOH	

## Data Availability

Data are contained within the article.

## Supplementary Materials

The following supporting information can be downloaded at https://www.mdpi.com/article/10.3390/molecules30214312/s1: Figure S1. Adsorption–desorption isotherms of the samples after the reaction. Figure S2. Adsorption–desorption isotherms of the samples after the reaction. Figure S3. TGA data of the Ce(mp)-2 sample after the reaction. Figure S4. TGA data of the Ce(mp)-3 sample after the reaction. Figure S5. TGA data of the Ce(mp)-4 sample after the reaction; TOF calculation procedure.

## Abbreviations

The following abbreviations are used in this manuscript:

BET	Brunauer–Emmett–Teller
DBD	Dielectric barrier discharge
EDX	Energy dispersive X-ray spectroscopy
SEM	Scanning electron microscopy
TGA	Thermogravimetric analysis
TPD	Temperature-programmed desorption
XPS	X-ray photoelectron spectroscopy
XRD	X-ray diffraction
XRF	X-ray fluorescent spectroscopy analysis
