# Supplementary material for: Plasma-Activated CO2 Dissociation to CO in Presence of CeO2 Mesoporous Catalysts
Source: Molecules. 2025 Nov 6;30(21):4312. doi: 10.3390/molecules30214312 (PMC12611055; doi:10.3390/molecules30214312)
Supplement: Supplementary file 1 [file molecules-30-04312-s001.zip › molecules-3961082-supplementary.pdf]

molecules

MDPI

Article

Plasma-activated CO_2 dissociation to CO in presence of CeO_2 mesoporous catalysts

Oleg V. Golubev[a]*, Alexey A. Sadovnikov[a], Anton L. Maximov[b]

[a]A.V. Topchiev Institute of Petrochemical Synthesis, Russian Academy of Sciences (TIPS RAS), 119991 Moscow, Russia
[b]Department of Chemistry, Lomonosov Moscow State University, 119991 Moscow, Russia
* Correspondence: golubev@ips.ac.ru

Supplementary Materials

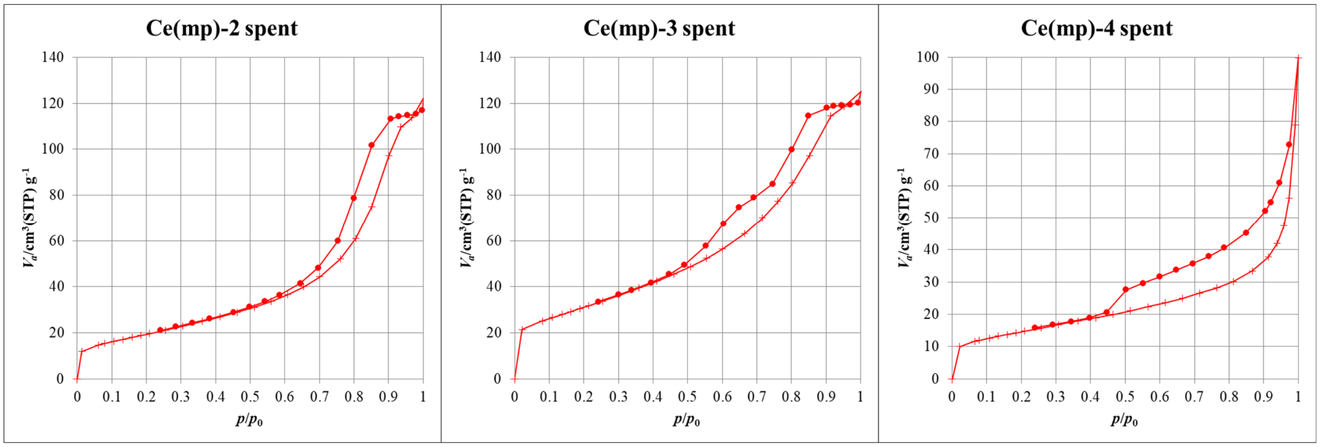

Figure S1. Adsorption-desorption isotherms of the samples after the reaction.

 https://doi.org/10.3390/xxxxx

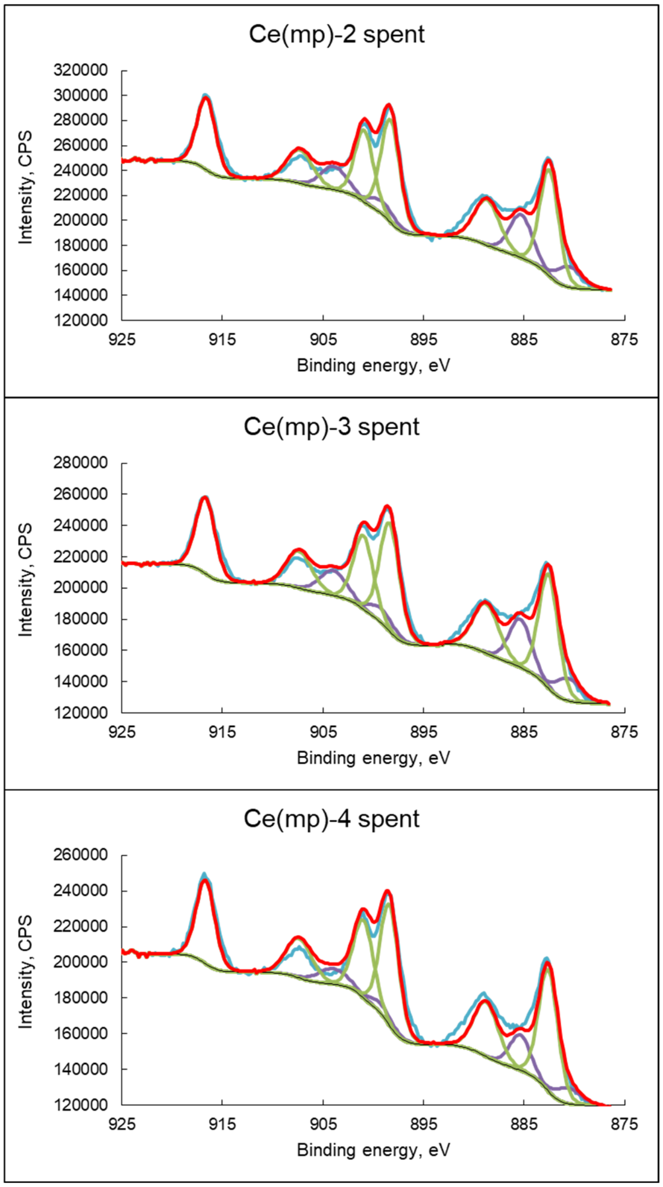

Figure S2. Adsorption-desorption isotherms of the samples after the reaction.

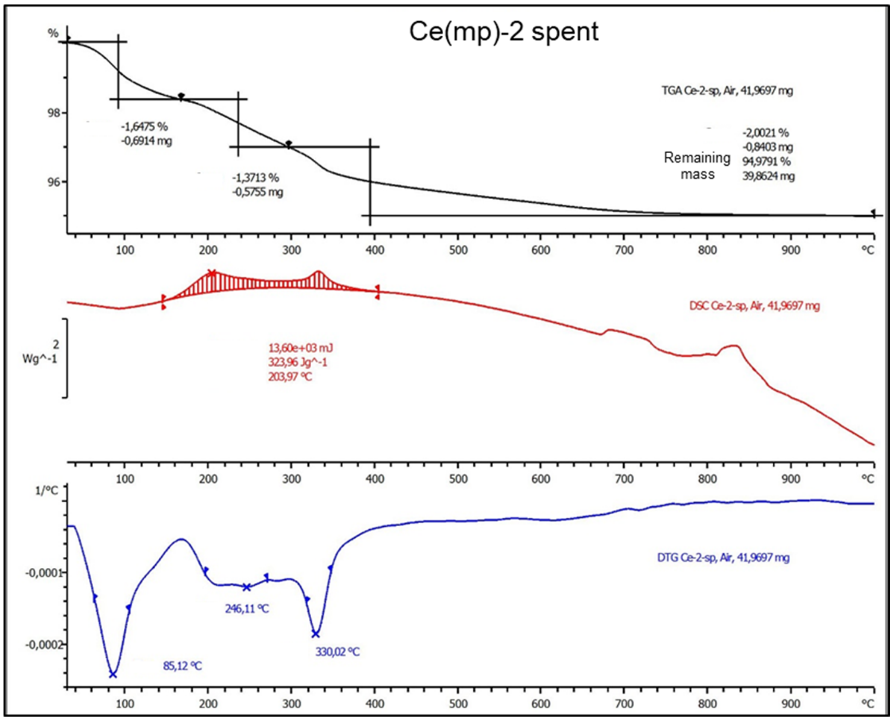

Figure S3. TGA data of the Ce(mp)-2 sample after the reaction.

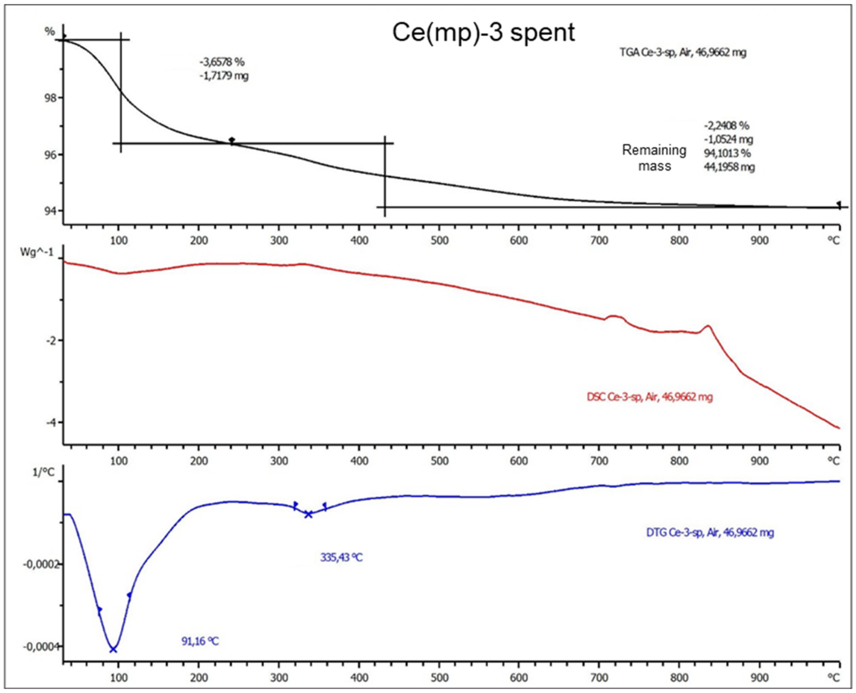

Figure S4. TGA data of the Ce(mp)-3 sample after the reaction.

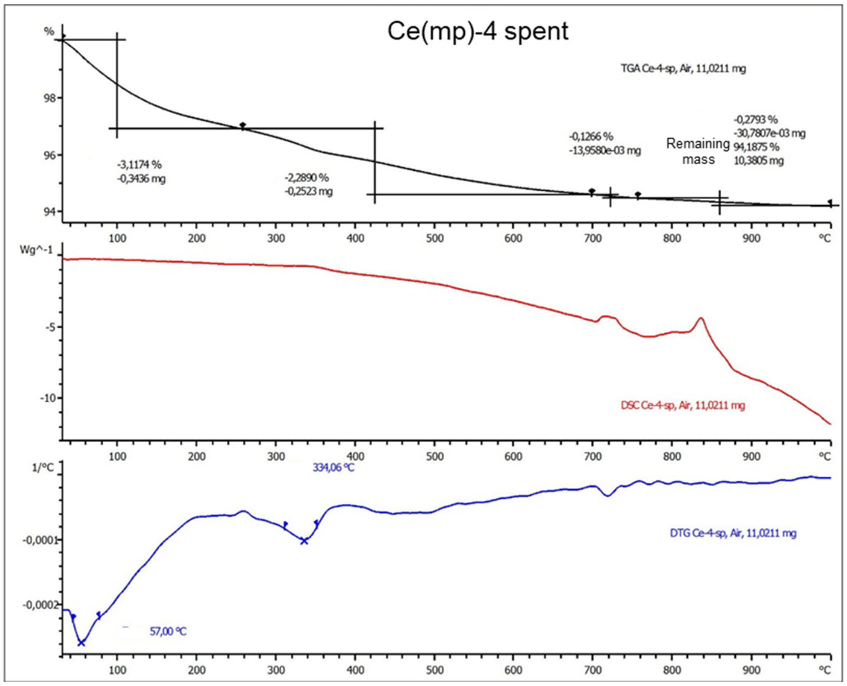

Figure S5. TGA data of the Ce(mp)-4 sample after the reaction.

TOF calculation.

In order to estimate the turnover frequency (TOF) of the catalysts, the several assumptions were made, thus, the obtained values may be referred to as "estimated TOF". For the current reaction, it was assumed that not all surface Ce atoms serve the "active sites", but only a Ce^{3+} fraction.

$$TOF(\%) = \frac{r(CO)}{\nu(Ce\ surface\ sites)} \times 100\% \qquad (S1)$$

where $r(CO)$ is the rate of CO formation (mol×s^{-1}), and $\nu(Ce\ surface\ atoms)$ is the quantity of the Ce active sites (mol×g^{-1}), which participate in the reaction. The rate of CO formation was calculated as:

$$r(CO)(\text{mol} \times s^{-1}) = F(CO_2)_{inlet} \times X(CO_2) \times S(CO) \qquad (S2)$$

where $F(CO_2)_{inlet}$ is the molar flowrate of CO_2 (mol×s^{-1}) and is fixed as 8.9×10^{-6} mol s^{-1}, $X(CO_2)$ – conversion of CO_2 during plasma-catalytic dissociation at 4 kV for each catalyst (%), $S(CO)$ – selectivity towards CO (%).

The quantity of the Ce active sites was calculated as:

$$\nu(Ce\ surface\ sites)(mol \times g^{-1}) = \frac{CeO_2\ (111) surface\ density}{N_A} \times S_{BET} \times \omega(Ce^{3+}) \qquad (S3)$$

Where $CeO_2\ (111) surface\ density$ is a value from the literature [1,2] and is 7.9 atoms per nm^2, N_A is the Avogadro constant (6.02214076×10^{23}×mol^{-1}), S_{BET} is the specific surface area of the sample, revealed with N_2 adsorption-desorption analysis, and $\omega(Ce^{3+})$ is the Ce^{3+} concentration, %, revealed from XPS analysis.

References

1. Nelson, N. C.; Wang, Z.; Naik, P.; Manzano, J. S.; Pruski, M.; Slowing, I. I. Phosphate modified ceria as a Brønsted acidic/redox multifunctional catalyst. *J. Mater. Chem. A* **2017**, *5*, 4455-4466. https://doi.org/10.1039/C6TA08703E
2. Madier, Y.; Descorme, C.; Le Govic, A. M.; Duprez, D. Oxygen mobility in CeO_2 and $Ce_xZr_{(1-x)}O_2$ compounds: study by CO transient oxidation and $^{18}O/^{16}O$ isotopic exchange. *J. Phys. Chem. B* **1999**, *103*, 10999–11006. https://doi.org/10.1021/jp991270a
